# Silkworm pupa oil attenuates acetaminophen‐induced acute liver injury by inhibiting oxidative stress‐mediated NF‐κB signaling

**DOI:** 10.1002/fsn3.1296

**Published:** 2019-11-28

**Authors:** Xingyao Long, Jiajia Song, Xin Zhao, Yu Zhang, Hongwei Wang, Xinqi Liu, Huayi Suo

**Affiliations:** ^1^ Beijing Advanced Innovation Center for Food Nutrition and Human Health Beijing Technology and Business University Beijing China; ^2^ College of Food Science Southwest University Chongqing China; ^3^ Chongqing Collaborative Innovation Center for Functional Food Chongqing University of Education Chongqing China

**Keywords:** acetaminophen, anti‐inflammatory, antioxidant, hepatic injury, silkworm pupa

## Abstract

Acetaminophen (APAP) overdose causes severe hepatotoxicity and acute liver failure. The current study aims to investigate the protection effects of silkworm pupa oil (SPO) against acute hepatic injury in APAP‐exposed Kunming mice. Our results showed that the liver index and the levels of serum alanine transaminase (ALT) and aspartate transaminase (AST) in mice subjected to APAP treatment were decreased by SPO. Supplement of SPO also restored hepatic histopathological alterations induced by APAP. The APAP‐induced increase in proinflammatory cytokines, including TNF‐α, IL‐6, and IL‐12, was reversed by SPO, which was mediated by the reduction of nuclear factor (NF)‐κB p65 expression and the increase in the expression of IκB‐α in liver tissue. Moreover, SPO inhibited APAP‐triggered oxidative stress by decreasing MDA level and increasing the activities of SOD and GSH‐Px. Collectively, SPO attenuated hepatic injury induced by APAP, which attributed to the suppression of oxidative stress‐mediated NF‐κB signaling. Our findings suggest that SPO supplementation may be potential strategy against acute hepatic injury.

## Graphical abstract

Silkworm pupa oil (SPO) reduced acetaminophen (APAP)‐induced oxidative stress, and thereby suppressed the activation of hepatic nuclear factor (NF)‐κB signaling and the production of proinflammatory cytokines. This ultimately attenuated APAP‐induced hepatic injury. Red solid line arrows mean alternations in mice with APAP treatment; arrows of blue dotted line mean alternations in APAP‐treated mice receiving SPO.

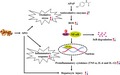

## 1 INTRODUCTION

The acute liver failure caused by drugs has become a major public health problem (Jiao, Xiao, Li, Liang, & Tang, 2018; Woolbright & Jaeschke, 2016). The overdose of acetaminophen, also called N‐acetyl‐p‐aminophenol (APAP), is considered as the leading cause of acute hepatic failure (Ghanem, Pérez, Manautou, & Mottino, 2016). APAP is a nonprescription drug used for the management of cough, pain, and hyperthermia. When used <4 g per day for adults, APAP is considered to be safe (Amin, Hashem, Alshehri, Awad, & Hassan, 2017). However, in overdose cases, such as the inadvertent or intentional ingestion, APAP leads to necrosis of a number of liver cells and acute hepatic failure (Antoine et al., 2013; Craig et al., 2011). Thus, an attempt to develop strategies against APAP‐induced hepatic injury is of great significance. Currently, the only FDA‐approved drug for APAP overdose is a potent antioxidant named N‐acetylcysteine (NAC; Khayyat, Tobwala, Hart, & Ercal, 2016). NAC promotes the resynthesis of cellular glutathione (GSH) under conditions of APAP overdose, which attenuates N‐acetyl‐p‐benzoquinone imine (NAPQI)‐mediated hepatic injury (Albano, Rundgren, Harvison, Nelson, & Moldéus, 1985; Corcoran, Racz, Smith, & Mitchell, 1985). Although oral or intravenous administration of NAC protects against APAP‐induced hepatotoxicity, NAC treatment has a high incidence of anaphylactic reactions (McNulty, Lim, Chandru, & Gunja, 2018; Sandilands & Bateman, 2009). Therefore, the development of effective ingredients with low adverse effects for APAP detoxification is clearly needed.

Some food‐derived functional components possess protective effects against APAP‐induced liver injury, which has received special attention. It is reported that *Opuntia* extracts reduce the levels of hepatic injury markers, including transaminase and alkaline phosphatase, and reverse APAP‐induced depletion of liver GSH and histological changes of liver (González‐Ponce et al., 2016). Baicalein pretreatment enhances the levels of hepatic antioxidant enzymes and alleviates the elevation of inflammatory cytokines and liver injury in APAP‐exposed mice (Zhou et al., 2019). Dietary unsaturated fatty acids have received extensive attention because of their broad therapeutic and culinary values. Supplementation with unsaturated fatty acids contributes to the management of various diseases, such as cardiovascular disorders and cancers (Asif, 2015; Lee & Park, 2014). Silkworm pupa, the main by‐product of the silk industry, is used for the preparation of high‐quality oil (Tomotake, Katagiri, & Yamato, 2010; Wei, Liao, Zhang, Liu, & Jiang, 2009). The unsaturated fatty acids in silkworm pupa oil (SPO) account for approximately 70% of total fatty acids (Hu et al., 2017). SPO exhibits the superior activities for 2,2‐diphenyl‐1‐picrylhydrazyl radical scavenging and the suppression of lipid peroxidation and tyrosinase (Hu et al., 2017; Manosroi, Boonpisuttinant, Winitchai, Manosroi, & Manosroi, 2010). Furthermore, SPO reduces high‐cholesterol diet (HCD)‐induced elevation of serum lipids and oxidative stress in HCD‐fed rats (Zou et al., 2017). In our previous study, we found that SPO protected against gastric ulcer in mice with hydrochloric acid/ethanol treatment (Long et al., 2019). However, whether SPO attenuates APAP‐induced hepatic injury in mice needs to be further investigated.

In our study, the effects of SPO on the serum markers for liver injury and pathologic changes in liver tissue were investigated using APAP‐treated Kunming (KM) mice. The activation of hepatic nuclear factor (NF)‐κB signaling, as well as the production of inflammatory cytokines, was assessed. Moreover, the effects of SPO on oxidative stress were further analyzed.

## 2 MATERIALS AND METHODS

### 2.1 Materials

Silkworm pupa oil was purchased from Harbin Essen Biotechnology. The fatty acid composition of SPO was reported in our previous study (Long et al., 2019). The antibody to IκB‐α was from Santa Cruz. The primary antibodies for β‐actin and NF‐κB p65, and anti‐mouse/rabbit secondary antibodies for Western blot were from Thermo Fisher Scientific.

### 2.2 Animal experiments

The 7‐week‐old male KM mice were supplied by Animal Experimental Center of Chongqing Medical University. They were given sufficient food and water and maintained under controlled environmental conditions (temperature of 25 ± 2°C, 12:12 hr light/dark cycle). These animals were divided into five groups: control (group 1); APAP (group 2); APAP plus positive drug silymarin (SLM; group 3); APAP plus low‐dosage SPO (group 4); and APAP plus high‐dosage SPO (group 5). The mice from groups 1 and 2 were orally gavaged with physiological saline once daily, while the mice from groups 3, 4, and 5 were administrated 100 mg/kg body weight (BW) of SLM, 3.75 and 7.50 ml/kg BW of SPO, respectively. After 2 weeks, all the mice were fasted overnight, and the mice from groups 2, 3, 4, and 5 were injected with 500 mg/kg BW of APAP intraperitoneally. After 16 hr, all the mice were euthanized, and the collection of blood and liver tissues was performed. The liver index was calculated as liver weight divided by the corresponding BW of mice.

### 2.3 Measurement of hepatic injury markers

The blood samples were centrifuged at 1,500 *g* for 10 min for serum production. The determinations of alanine transaminase (ALT) and aspartate transaminase (AST) were carried out based on commercial kits (Nanjing Jiancheng Bioengineering Institute).

### 2.4 Histological analysis

Fresh hepatic tissue was fixed in 10% formalin and then embedded in paraffin. The 5 µm of hepatic tissue sections was prepared, followed by the procedure of hematoxylin and eosin (HE) staining.

### 2.5 Inflammatory cytokines assay

The contents of serum tumor necrosis factor (TNF)‐α, interleukin (IL)‐6, IL‐12, and IL‐10 were assayed by commercial kits obtained from Cloud‐Clone Corp.

### 2.6 Determination of oxidative stress

The levels of serum malondialdehyde (MDA), superoxide dismutase (SOD), and glutathione peroxidase (GSH‐Px) were determined by commercial kits (Solarbio).

### 2.7 Analysis of mRNA expression

Total RNA was isolated from liver tissue using TRIzol reagent (Thermo Fisher Scientific), and reverse‐transcripted to cDNA by Revert‐Aid™ first‐strand cDNA synthesis kit (Thermo Fisher Scientific). Quantitative real‐time polymerase chain reaction was performed using Master Mix (Thermo Fisher Scientific) in StepOnePlus™ Real‐Time System (Thermo Fisher Scientific). The 2^−ΔΔT^ method was used for the calculation of the relative mRNA expression. The sequences of primers for qRT‐PCR were as follows: GAPDH forward, 5′‐AGGTCGGTGTGAACGGATTTG‐3′; reverse, 5′‐GGGGTCGTTGATGGCAACA‐3′; IκB‐α forward, 5′‐TGAAGGACGAGGAGTACGAGC‐3′; reverse, 5′‐TGCAGGAACGAGTCTCCGT‐3′; NF‐κB forward, 5′‐ATGGCAGACGATGATCCCTAC‐3′; reverse, 5′‐CGGAATCGAAATCCCCTCTGTT‐3′.

### 2.8 Western blot

The hepatic proteins were extracted using RIPA lysis buffer supplemented with phenylmethylsulfonyl fluoride (Solarbio). The extracted proteins were loaded on sodium dodecyl sulfate polyacrylamide gel for electrophoresis separation, and then, the proteins on the gel were transferred onto polyvinylidene fluoride (PVDF) membrane (Thermo Fisher Scientific) in a transfer buffer. The PVDF membrane containing proteins was blocked by 5% nonfat milk powder, followed by incubation with primary and secondary antibodies according to the recommended methods from manufacturers. The SuperSignal West Pico chemiluminescent substrate (Thermo Fisher Scientific) was used for the development of protein bands. The quantitative analysis of bands was carried out using NIH ImageJ.

### 2.9 Statistical analysis

Data were presented as mean ± standard deviations (*SD*). Experimental differences were assessed by one‐way ANOVA and Duncan's multivariate using SPSS version 22.0 (IBM). A *p* value of <.05 was considered significant.

## 3 RESULTS

### 3.1 SPO reduced liver index and the levels of serum transaminases in mice with APAP treatment

**Figure 1:**
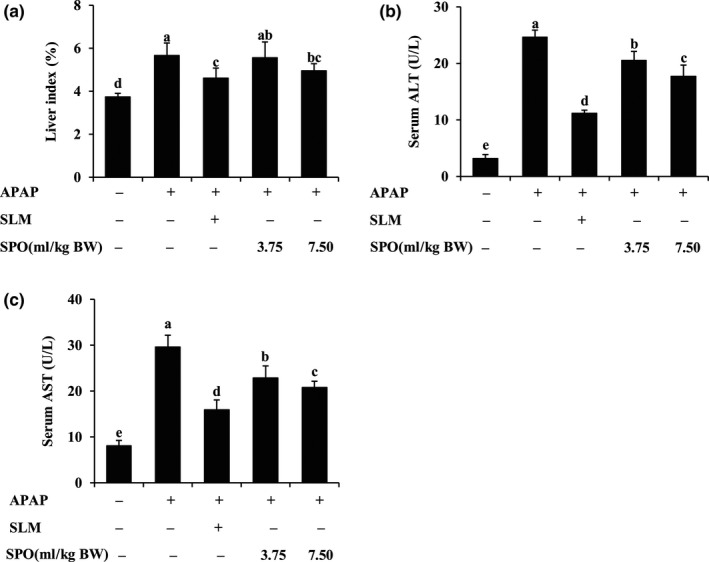
Effects of silkworm pupa oil on liver index and the levels of serum transaminases. (a) Liver index; (b) Serum alanine aminotransferase (ALT) level; (c) Serum aspartate aminotransferase (AST) level. APAP, acetaminophen; SLM, positive drug silymarin; SPO, silkworm pupa oil. Values presented are mean ± standard deviations (*SD*) of eight mice. Values with different letters are significantly different (*p* < .05)

To investigate the protection effects of SPO against hepatic injury induced by APAP, the liver index and the levels of serum transaminases were determined. Compared with control mice, APAP treatment significantly increased liver index in mice (*p* < .05; Figure 1). However, the increase in liver index induced by APAP was reduced by the pretreatment of SLM and high dosage of SPO (*p* < .05). In addition, as shown in Figure 1b,c, the levels of serum transaminases, including ALT and AST, in APAP‐exposed mice were increased in comparison with control mice (*p* < .05), while SLM and SPO markedly decreased the levels of these serum transaminases (*p* < .05). Moreover, SPO displays a dose‐dependent effect on the suppression of serum transaminases in mice subjected to APAP treatment. These results showed that SPO reduced APAP‐induced increase in liver index and the levels of serum transaminases, suggesting that SPO possessed protective effects against hepatic injury induced by APAP.

### 3.2 SPO alleviated APAP‐induced liver histopathology abnormalities

**Figure 2:**
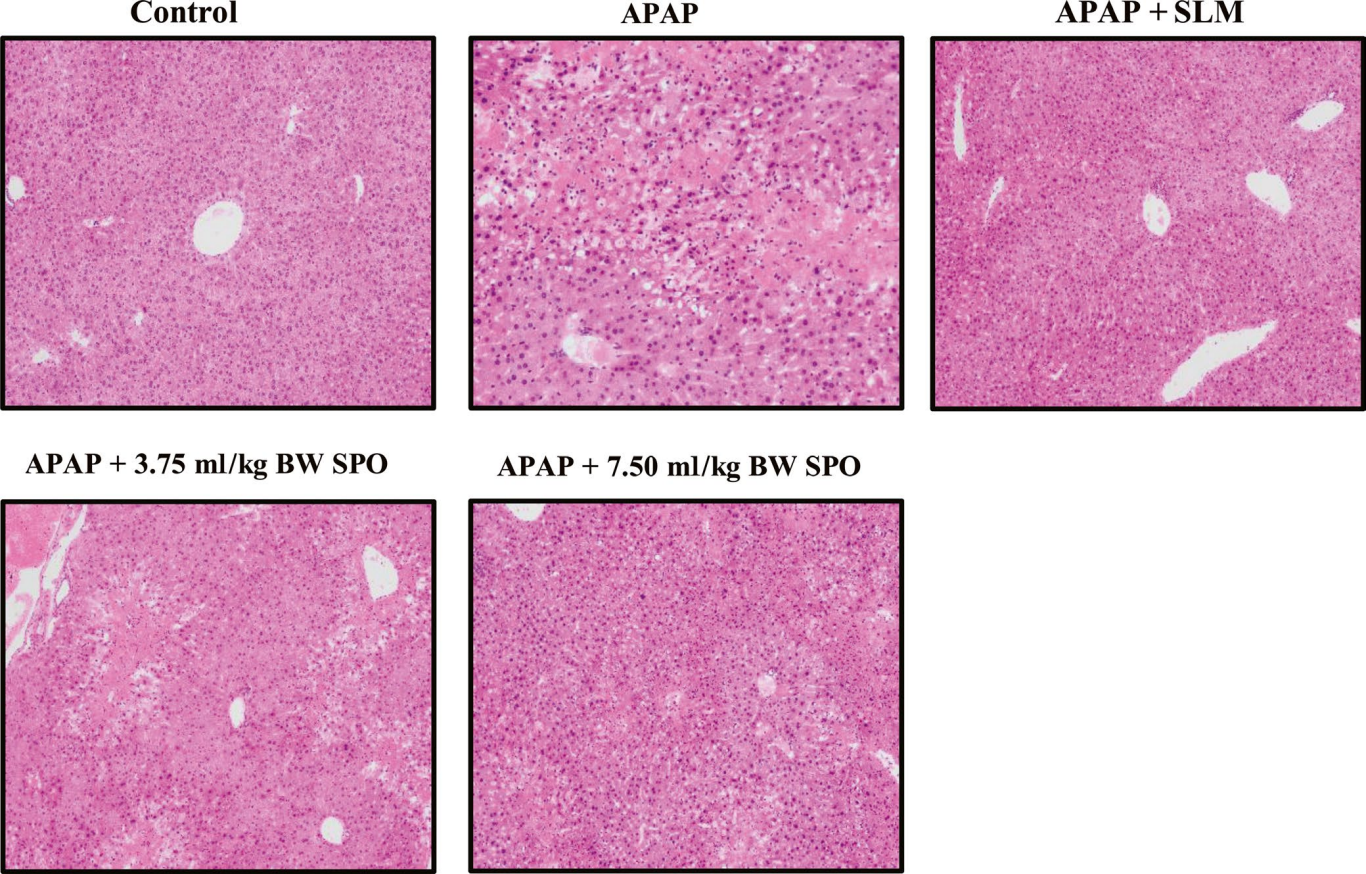
Effects of silkworm pupa oil on hepatic histology (original magnification 100×). APAP + 3.75 ml/kg BW SPO, acetaminophen‐treated mice received 3.75 ml/kg body weight of silkworm pupa oil; APAP + 7.50 ml/kg BW SPO, acetaminophen‐treated mice received 7.50 ml/kg body weight of silkworm pupa oil; APAP + SLM, acetaminophen‐treated mice received positive drug silymarin; APAP, acetaminophen treatment; Control, no treatment. *n* = 6

To further evaluate the hepatic protective effects of SPO, the liver histopathology of APAP‐treated mice was analyzed. As shown in Figure 2, hepatocytes in control mice were separated by blood sinusoids and arranged around central vein, which showed normal liver histology. APAP treatment damaged liver structure, as indicated by intrahepatic hemorrhage and intense cytoplasmic vacuolation of hepatocytes. However, supplementation of SPO dose‐dependently restored hepatic morphological changes. The attenuation of abnormalities in hepatic structure was also observed in SLM‐treated mice. Collectively, SPO has the ability of protecting against APAP‐induced liver damage.

### 3.3 SPO inhibited APAP‐induced inflammation in mice

**Figure 3:**
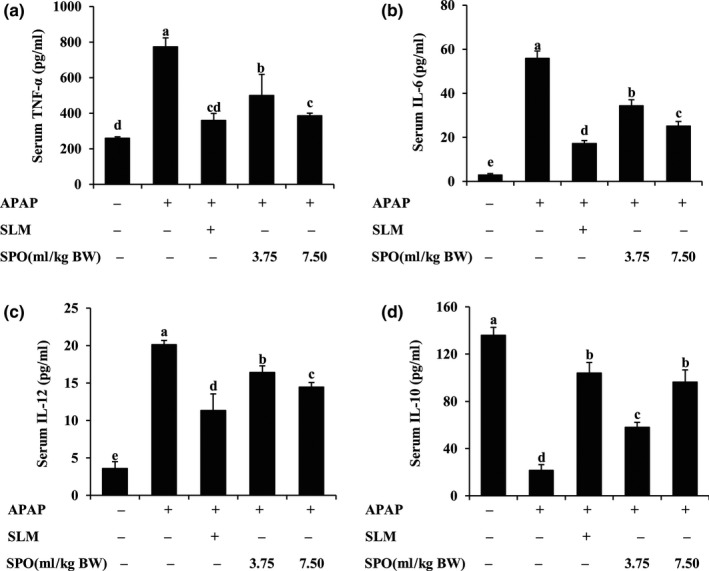
Effects of silkworm pupa oil on the contents of serum inflammatory cytokines. (a) Tumor necrosis factor (TNF)‐α level; (b) Interleukin (IL)‐6 level; (c) IL‐12 level; (d) IL‐10 level. APAP, acetaminophen; SLM, positive drug silymarin; SPO, silkworm pupa oil. Values presented are mean ± standard deviations (*SD*) of eight mice. Values with different letters are significantly different (*p* < .05)

To evaluate the inhibitory effects of SPO on inflammation induced by APAP, the contents of serum inflammatory cytokines were assayed. Compared with control mice, APAP mice displayed significantly higher contents of serum TNF‐α, IL‐6, and IL‐12 (Figure 3a,b,c; *p* < .05). The administration of SPO with different dosages, as well as SLM, effectively decreased the levels of these inflammatory cytokines in APAP‐treated mice (*p* < .05). In addition, Figure 3d showed that the serum IL‐10 level was reduced by only APAP treatment. This decrease in the level of IL‐10 in APAP‐treated mice was significantly reversed by SLM and SPO administration (*p* < .05). Moreover, we found that high‐dose SPO treatment exhibited similar effects on the contents of serum TNF‐α and IL‐10 as SLM (*p* > .05). Our data demonstrated that SPO inhibited inflammatory response in APAP‐treated mice, which prevented against hepatic toxicity induced by APAP.

### 3.4 SPO inactivated NF‐κB signaling in APAP‐treated mice

**Figure 4:**
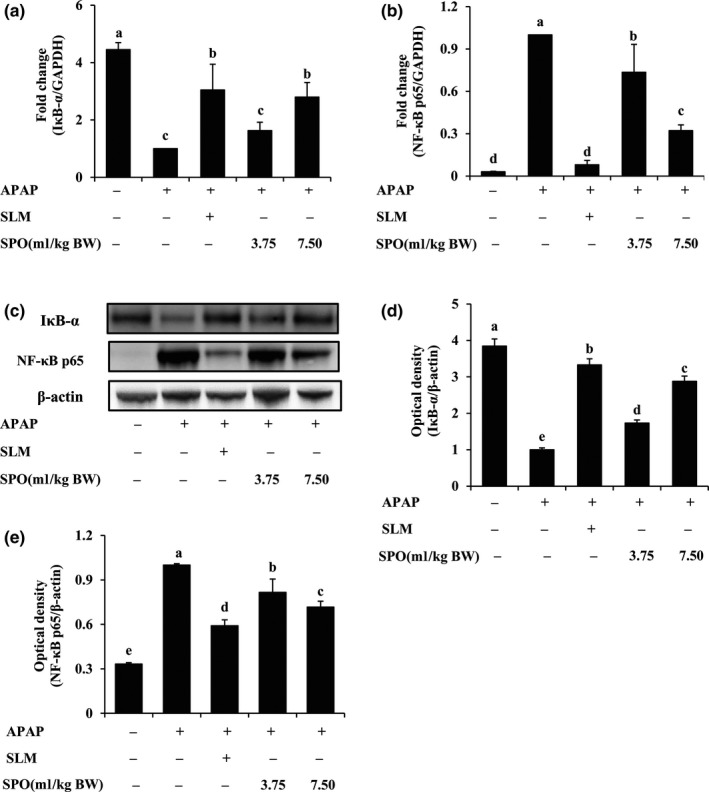
Effects of silkworm pupa oil on hepatic nuclear factor (NF)‐κB signaling. (a) NF‐κB inhibitor IκB‐α mRNA expression; (b) NF‐κB p65 mRNA expression; (c) A representative blot for IκB‐α and NF‐κB p65; (d) Densitometric quantification of IκB‐α; (e) Densitometric quantification of NF‐κB p65. APAP, acetaminophen; SLM, positive drug silymarin; SPO, silkworm pupa oil. Values presented are mean ± standard deviations (*SD*) of six mice. Values with different letters are significantly different (*p* < .05)

To elucidate the potential mechanisms underlying SPO‐mediated reduction in inflammatory response, the activation of hepatic NF‐κB signaling was assessed. As shown in Figure 4a,c,d, the expression of hepatic IκB‐α mRNA and protein was markedly reduced by APAP treatment (*p* < .05). However, supplement of SPO dose‐dependently reversed this decrease in hepatic IκB‐α expression in APAP‐treated mice (*p* < .05). Similarly, SLM increased IκB‐α expression in liver tissue of mice with APAP treatment. Furthermore, the expression of NF‐κB p65 at gene and protein level was markedly elevated in APAP‐treated mice in comparison to control mice without treatment (*p* < .05), while the elevation effects of APAP on hepatic NF‐κB p65 expression were blocked by SLM and SPO (*p* < .05; Figure 4b,c,e). These results indicated that SPO alleviated APAP‐induced inflammatory response and hepatotoxicity by the inactivation of NF‐κB signaling.

### 3.5 SPO attenuated oxidative stress in APAP‐treated mice

In order to further explore the mechanisms by which SPO suppressed APAP‐induced inflammatory response, the effects of SPO on oxidative stress were investigated. Table 1 showed that APAP treatment markedly increased the serum MDA level, which indicates that the reactive oxygen species (ROS) and related lipid peroxidation occurred in APAP‐treated mice. However, the high level of serum MDA induced by APAP was dose‐dependently decreased by SPO supplementation (*p* < .05). Additionally, in comparison with control mice, the levels of serum SOD and GSH‐Px were diminished in APAP‐treated mice (*p* < .05). However, SPO dose‐dependently reversed APAP‐induced decrease in the activities of these antioxidases (*p* < .05). As expected, SLM also reduced the levels of oxidative stress in mice with APAP treatment. Altogether, these results demonstrated that SPO enhanced antioxidant ability and inhibited oxidative stress in APAP‐treated mice, which contributes to the attenuation of hepatic inflammation and injury.

**Table 1:** Effects of silkworm pupa oil on the levels of malondialdehyde (MDA), superoxide dismutase (SOD), and glutathione peroxidase (GSH‐Px)

Group	MDA (mol/ml)	SOD (U/ml)	GSH‐Px (U/ml)
Control	2.15 ± 0.07^e^	117.35 ± 2.11^a^	85.55 ± 1.83^a^
APAP	15.69 ± 1.32^a^	55.42 ± 0.37^e^	13.67 ± 0.62^e^
APAP + SLM	4.67 ± 0.23^d^	109.33 ± 2.79^b^	69.73 ± 1.11^b^
APAP + 3.75 ml/kg BW SPO	10.16 ± 0.65^b^	70.49 ± 1.35^d^	25.36 ± 1.67^d^
APAP + 7.50 ml/kg BW SPO	8.75 ± 0.32^c^	97.56 ± 2.04^c^	49.96 ± 2.05^c^

Note

Values presented are mean ± standard deviations (*SD*) of eight mice. Values with different letters are significantly different (*p* < .05).

Abbreviations: APAP + 3.75 ml/kg BW SPO, acetaminophen‐treated mice received 3.75 ml/kg body weight of silkworm pupa oil; APAP + 7.50 ml/kg BW SPO, acetaminophen‐treated mice received 7.50 ml/kg body weight of silkworm pupa oil; APAP + SLM, acetaminophen‐treated mice received positive drug silymarin; APAP, acetaminophen treatment; Control: no treatment.

## 4 DISCUSSION

Overdose of APAP is well known to cause severe hepatic injury, which can progress to acute liver failure. Serum ALT and AST are common biomarkers for detection of hepatic injury, and the elevated levels of serum transaminases have been attributed to damaged liver (Goorden, Buffart, Bakker, & Buijs, 2013; Rasool et al., 2019). It is reported that APAP overdose leads to hepatic histopathological lesions, such as cell swelling and necrosis, which increases the contents of serum ALT and AST (Omidi, Riahinia, Torbati, & Behdani, 2014; Uchida et al., 2017; Xie, Jiang, Wang, Zhang, & Melzig, 2016). As expected, in our study, APAP‐treated mice showed the elevated levels of serum transaminases, as well as liver swelling and increased hepatic index. However, the hepatomegaly and high levels of serum transaminases were attenuated by SPO administration. These preliminary observations indicate that SPO protected against liver injury induced by APAP.

It is noteworthy that hepatic injury is considered to be associated with the elevated levels of proinflammatory cytokines. TNF‐α plays a decisive role in the progression of hepatic injury induced by APAP (Devkar et al., 2016). The protein expression of hepatic TNF‐α is increased 4 hr after APAP treatment and gets to the highest level at 10 hr (Ishida et al., 2004). The increased level of TNF‐α makes TNF‐α to bind to its receptor TNF‐α receptor 1 (TNF‐R1), which drives the activation of hepatic apoptosis and necrosis signaling (Chao, Wang, & Ding, 2017; Filliol et al., 2016). Moreover, TNF‐α can induce the release of other proinflammatory cytokines. The levels of IL‐6 and IL‐12 are increased in primary human hepatocytes and mice with APAP treatment (Cho et al., 2015; Kim et al., 2017). Inhibition of proinflammatory cytokines production attenuates inflammation‐mediated hepatocyte injury following APAP toxicity (Devkar et al., 2016; Hussan et al., 2015). By contrast, IL‐10, a well‐established anti‐inflammatory cytokine, is shown to protect against APAP toxicity. The susceptibility of halothane‐induced hepatic injury is increased in IL‐10 knockout mice, while the supplement of IL‐10 prevents susceptible mice from hepatic damage (Feng et al., 2009). Furthermore, the activation of NF‐κB signaling can induce the production of proinflammatory cytokines and hepatic inflammation. NF‐κB subunits p50/p65 are bound to the inhibitory protein IκBα and exist in cytosol as an inactive form (Evans, Rodino, Adcox, & Carlyon, 2018; Whitman & Barber, 2015). Upon stimulation, IκBα will be phosphorylated and subsequently degraded, and then, unbonded NF‐κB initiates the transcription of proinflammatory cytokines. Hence, the maintenance of hepatocellular NF‐κB/IκBα stability may contribute to the attenuation of hepatic injury. It is reported that bioactive components, such as α‐mangostin and isoquercitrin, show hepatoprotective effects in APAP‐treated mice, which partially attributes to the inactivation of NF‐κB signaling (Fu et al., 2018; Xie, Wang, Chen, Zhang, & Melzig, 2016). In line with these findings, our results suggest that SPO significantly inactivated NF‐κB signaling in mice subjected to APAP treatment, which reduced the production of proinflammatory cytokines, and eventually prevented inflammation‐mediated hepatic injury.

Acetaminophen‐induced oxidative stress contributes to inflammation and the pathology process of acute hepatic injury. APAP undergoes conversion to a toxic metabolite NAPQI by cytochrome P450, which leads to NAPQI‐GSH formation, the rapid depletion of liver GSH, and the excessive formation of mitochondrial ROS (Antoine, Williams, & Park, 2008; Khodayar, Kalantari, Khorsandi, Rashno, & Zeidooni, 2018). Except for the reduction of GSH, APAP also decreases the activities of antioxidative enzymes to further enhance oxidative stress (O'Brien et al., 2000). Moreover, the elevated levels of ROS activate NF‐κB signaling and upregulate the expression of inflammatory mediators, which is associated with hepatic inflammation and injury (Chen, Hu, & Yin, 2016; Hong, Lee, Jung, Lee, & Hong, 2012). Inhibition of hepatic oxidative stress and inflammation attenuates hepatotoxicity induced by APAP (Ding et al., 2016; Huang et al., 2017). In our study, SPO reduced the level of MDA and increased the activities of antioxidases in APAP‐treated mice, indicating that SPO attenuated APAP‐induced oxidative stress via inhibiting ROS‐mediated lipid peroxidation and improving antioxidant defenses. The reduction effects of SPO on oxidative stress may contribute to the inhibition of hepatic inflammation and the protection against APAP hepatotoxicity. Our previous study reported that SPO contained 69.73% unsaturated fatty acid, and the linolenic acid and oleic acid accounted for the majority of unsaturated fatty acid (Long et al., 2019). Omega‐3 polyunsaturated fatty acids inhibit NF‐κB‐mediated inflammation and have potent protective effects against hepatotoxicity induced by APAP overdose (Feng et al., 2018). The supplement of olive oil rich in oleic acid and palmitoleic acid decreases the levels of liver fibrotic markers in carbon tetrachloride‐induced liver fibrosis (Chiang & Chao, 2018). Based on these researches, it could be speculated that the high levels of unsaturated fatty acid in SPO may be responsible for its anti‐hepatic injury effects.

## 5 CONCLUSION

**Figure 5:**
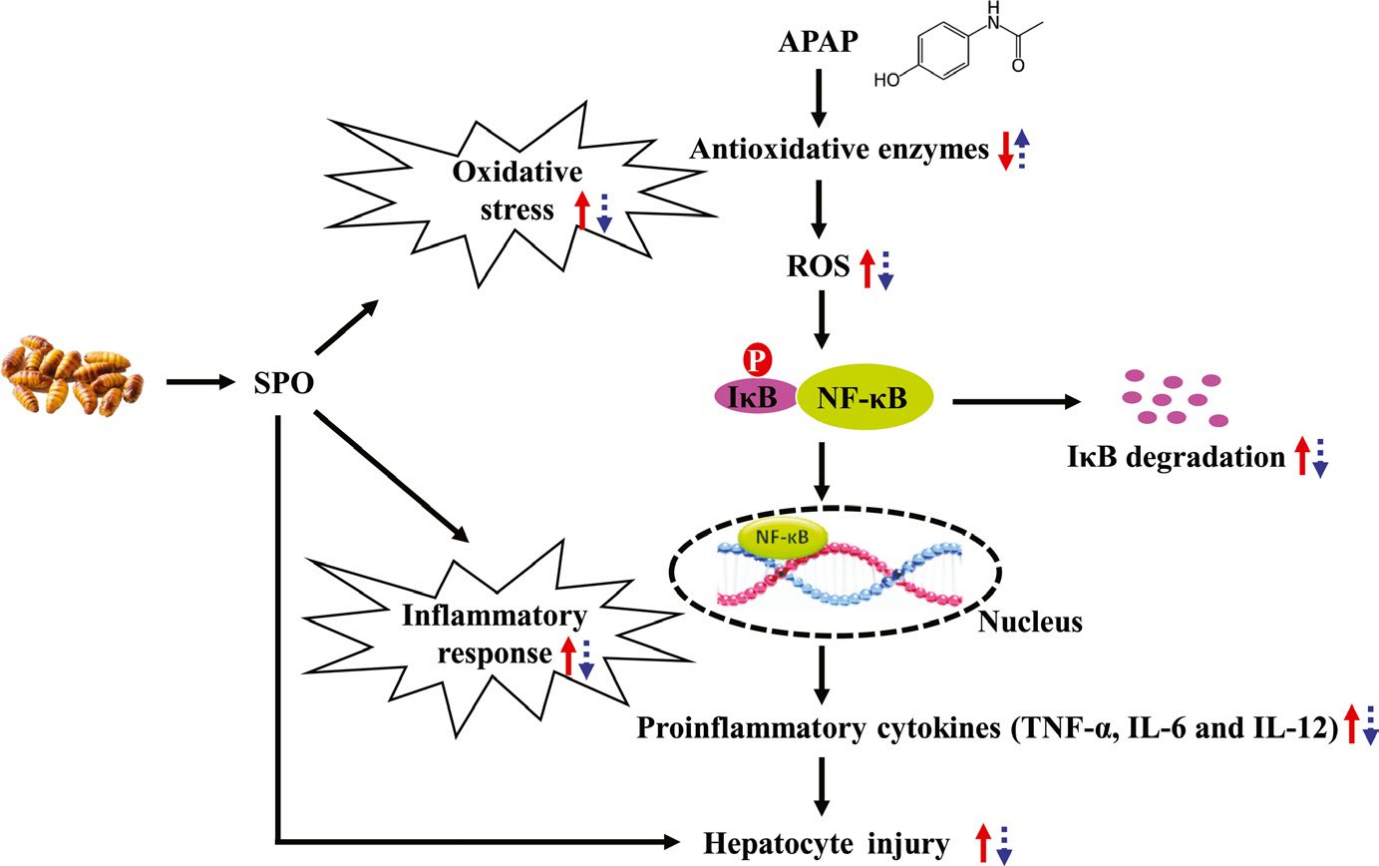
Protection effects of silkworm pupa oil (SPO) against hepatic injury induced by acetaminophen (APAP). SPO reduced APAP‐induced oxidative stress, and thereby suppressed the activation of hepatic nuclear factor (NF)‐κB signaling and the production of proinflammatory cytokines. This ultimately attenuated APAP‐induced hepatic injury. Red solid line arrows mean alternations in mice with APAP treatment; arrows of blue dotted line mean alternations in APAP‐treated mice receiving SPO

In summary, SPO reduced the liver index and the levels of serum transaminases and improved histological changes in APAP‐treated mice, suggesting that SPO protected against APAP‐induced hepatic injury. As depicted in Figure 5, these protection effects of SPO against hepatic injury in APAP‐exposed mice were involved in the inactivation of NF‐κB signaling and the decrease in the production of proinflammatory cytokines. Furthermore, SPO attenuated APAP‐induced oxidative stress, which alleviates hepatic inflammation and injury. Our results indicate that SPO may be a functional agent for the management of hepatic injury.

## CONFLICT OF INTEREST

The authors declare no conflict of interest.

## ETHICAL APPROVAL

The protocol for animal experiments was approved by the Animal Ethics and Experimental Committee of Chongqing Collaborative Innovation Center for Functional Food (Chongqing, China).
